# Plasticity in leaf‐level water relations of tropical rainforest trees in response to experimental drought

**DOI:** 10.1111/nph.13927

**Published:** 2016-03-22

**Authors:** Oliver Binks, Patrick Meir, Lucy Rowland, Antonio Carlos Lola da Costa, Steel Silva Vasconcelos, Alex Antonio Ribeiro de Oliveira, Leandro Ferreira, Bradley Christoffersen, Andrea Nardini, Maurizio Mencuccini

**Affiliations:** ^1^ School of GeoSciencesUniversity of EdinburghEdinburghEH9 3FEUK; ^2^ Research School of BiologyAustralian National UniversityCanberraACT2601Australia; ^3^ Centro de GeosciênciasUniversidade Federal do ParáBelém66075‐110Brazil; ^4^ EMBRAPA Amazônia OrientalBelém66095‐903Brazil; ^5^ Museu Paraense Emílio GoeldiBelém66077‐830Brazil; ^6^ Earth and Environmental ScienceLos Alamos National LaboratoryLos AlamosNM87545USA; ^7^ Dipartimento di Scienze della VitaUniversitá di TriesteVia L. Giorgieri 1034127TriesteItaly; ^8^ ICREA at CREAF08193Cerdanyola del VallésSpain

**Keywords:** Amazon rainforest, experimental drought, leaf anatomy, osmotic adjustment, plasticity, water relations

## Abstract

- The tropics are predicted to become warmer and drier, and understanding the sensitivity of tree species to drought is important for characterizing the risk to forests of climate change. This study makes use of a long‐term drought experiment in the Amazon rainforest to evaluate the role of leaf‐level water relations, leaf anatomy and their plasticity in response to drought in six tree genera.
- The variables (osmotic potential at full turgor, turgor loss point, capacitance, elastic modulus, relative water content and saturated water content) were compared between seasons and between plots (control and through‐fall exclusion) enabling a comparison between short‐ and long‐term plasticity in traits. Leaf anatomical traits were correlated with water relation parameters to determine whether water relations differed among tissues.
- The key findings were: osmotic adjustment occurred in response to the long‐term drought treatment; species resistant to drought stress showed less osmotic adjustment than drought‐sensitive species; and water relation traits were correlated with tissue properties, especially the thickness of the abaxial epidermis and the spongy mesophyll.
- These findings demonstrate that cell‐level water relation traits can acclimate to long‐term water stress, and highlight the limitations of extrapolating the results of short‐term studies to temporal scales associated with climate change.

## Introduction

The Amazon accounts for half the world's tropical rainforest (Fritz *et al*., 2003), contains *c*. 123 ± 31 pg of carbon in woody biomass (Malhi *et al*., 2006; Saatchi *et al*., 2007; FAO, 2010), contributes over 10% of the world's biodiversity (Da Silva *et al*., 2005; Lewinsohn & Prado, 2005) and is suggested to influence rainfall patterns as far away as Asia (Lawrence & Vandecar, 2015). Many of the ecosystem functions and services carried out by the forests of the Amazon basin are dependent on its hydrologic regime (Boisier *et al*., 2015). Yet, Earth system models have been used to suggest that the hydrology of the Amazon may change drastically under future climate change scenarios through increases in dry season length, long‐term soil drying, and increased frequency and intensity of drought events (Christensen *et al*., 2013; Fu *et al*., 2013; Reichstein *et al*., 2013; Boisier *et al*., 2015). Such shifts in climate may result in higher tree mortality (Phillips *et al*., 2009; Allen *et al*., 2010), threaten biodiversity and increase the possibility of climate feedbacks, the magnitude and direction of which remain uncertain. Currently, vegetation models used to represent the dynamic response to climate in Earth system models (dynamic global vegetation models (DGVMs)) lack the capability to predict ecological responses to drought within tropical forests reliably (Powell *et al*., 2013; Meir *et al*., 2015a), in part as a consequence of poor representation of how soil water stress influences leaf‐scale processes (Rowland *et al*., 2015b). To improve such representations, a greater empirical understanding of how soil water stress impacts leaf‐level processes is necessary.

According to the cohesion‐tension theory (Dixon & Joly, 1895), water moves down a free energy gradient (water potential (Ψ)) from soil to the leaves (following a pressure gradient along the xylem). For a plant to maintain its transpiration stream during drought, the leaves must be able to generate and sustain lower Ψ than the soil (Bowman & Roberts, 1985). The presence of solutes in the symplast (usually represented as osmotic potential (Ψ_π_), with more negative values indicating higher solute concentration) enables leaves to reach lower Ψ than the soil while maintaining turgor pressure. Thus, a lower osmotic potential enables a plant to function while drawing water from drier soil (Bowman & Roberts, 1985). Consequently, both osmotic potential at full turgor ($\Psi_{\pi }^{o}$) and the water potential at turgor loss point ($\Psi_{\pi }^{\mathrm{tlp}}$) are good predictors of plant sensitivity to drought stress (Bartlett *et al*., 2012). Turgor loss point is influenced by both the bulk modulus of elasticity (ε; the difference in turgor per unit relative change in cell volume) and $\Psi_{\pi }^{o}$, which appears to be the stronger determinant (Lenz *et al*., 2006; Bartlett *et al*., 2012). Additional water relation parameters derived from pressure–volume (PV) curves, for example capacitance, relative water content at $\Psi_{\pi }^{\mathrm{tlp}}$ and saturated water content, can also affect the drought sensitivity of a plant.

Osmotic adjustment to seasonal water stress is common and has been the focus of much research (see Bartlett *et al*., 2012 for a review). However, few, if any, studies have directly addressed the question of how the capacity for seasonal adjustment equips species to cope with long‐term shifts in water availability. Is there a physiological limit to osmotic adjustment determined by typical dry season water availability? Do species showing greater seasonal variability in water relations stand a better chance of coping with long‐term climate changes? Understanding the variation and plasticity of leaf tissue‐level parameters is essential to answering these questions and determining the ecosystem‐level response to environmental change.

Recent evidence suggests that tissues within leaves may be functionally ‘sequestered from one another’ (Rockwell *et al*., 2014; Buckley, 2015; Buckley *et al*., 2015). Leaf tissues are likely to experience different levels of hydration during transpiration (Rockwell *et al*., 2014; Buckley *et al*., 2015), and may be hydraulically compartmentalized (Nardini *et al*., 2010; Blackman & Brodribb, 2011; Canny *et al*., 2012). Given the evidence that the palisade mesophyll maintains turgor during transpiration (Canny *et al*., 2012; Buckley *et al*., 2015), we hypothesize that it may have a more negative osmotic potential than other cell layers. If that were the case, one might predict a correlation to emerge between palisade relative thickness and tissue‐level osmotic potential. Furthermore, Canny *et al*. (2012) also observed that spongy mesophyll cells ‘easily lose water’ compared with the palisade matrix cells, so we suggest that the spongy mesophyll acts as a hydraulic buffer. A relationship could thus be postulated between spongy mesophyll volume (excluding airspaces) and tissue‐level capacitance (Canny *et al*., 2012). Linking drought stress vulnerability with pressure volume traits and leaf anatomy could both strengthen the current understanding of leaf function and facilitate the identification of traits indicative of drought sensitivity or tolerance.

This study aimed to test whether tropical rainforest species can acclimate to changes in water availability on both a short time‐scale, represented by seasonal differences, and a long time‐scale, using a long‐term (> 12 yr) through‐fall exclusion experiment (TFE) in the Caxiuanã National Forest Reserve, State of Para, in Brazil. We correlated tissue‐level pressure volume parameters with leaf anatomical traits for indications of whether particular cell types contribute disproportionately to some PV traits, thus examining linkages between tissue form and function. The following hypotheses were tested.

1. Acclimation to long‐term soil moisture deficit results in greater osmotic adjustment and changes in elastic modulus than does acclimation to seasonal differences in soil moisture availability. Thus, osmotic potential at full turgor and turgor loss point are expected to be more negative, and elastic modulus more positive, in response to the long‐term drought treatment than in response to dry season changes
2. Drought‐resistant taxa show greater seasonal osmotic adjustment than drought‐sensitive taxa.
3. Palisade volume per unit leaf area correlates negatively with osmotic potential at full turgor and turgor loss point, suggesting higher solute concentration in this tissue. Spongy mesophyll volume per unit area correlates positively with capacitance, indicating a role as a water storage site.

In summary, this study aimed to determine how leaf water relations parameters varied in response to changes in water availability that resulted from seasonal differences in rainfall and a long‐term field‐scale soil moisture reduction experiment in trees from the lowland Amazon rainforest. Changes in parameters attributable to seasonal variation in rainfall were compared with those arising from an experimentally imposed drought (soil moisture deficit) to explore the adaptive capacity of rainforest tree leaves. The PV parameters were modeled against the absolute and relative values of thickness and volume of the leaf tissues to provide an indication of whether hydraulic differences occur among cell layers, and to facilitate the identification of traits indicative of differential drought sensitivity.

## Materials and Methods

### Study site

The study was undertaken in the Caxiuanã National Forest Reserve in the eastern Amazon (1^o^43′S, 51^o^27′W). The site is situated in lowland *terra firme* rainforest 10–15 m above river level. The site has a mean temperature of *c*. 25°C, receives 2000–2500 mm of rainfall annually and has a dry season in which rainfall is < 100 mm per month between June and November. The soil is a yellow oxisol of 3–4 m depth, below which is a laterite layer 0.3–0.4 m thick (Fisher *et al*., 2007).

### Large‐scale through‐fall exclusion experiment (TFE)

The TFE is one hectare of rainforest in which canopy through‐fall has been reduced by *c*. 50% since January 2002 (Meir *et al*., 2015b). An artificial ‘roof’ was constructed from clear plastic panels and wooden guttering at a height of 1–2 m above the ground. The intercepted water is channeled down‐slope to a point > 50 m away from the TFE. Both the TFE and the nearby control plot are surrounded by trenches 1–2 m deep to prevent lateral subsurface flow of water into the study plots. The plots, both 1 ha, are divided into 10 m × 10 m subplots and the outermost subplots are excluded from the study to mitigate the potential for edge effects on tree growth. For further details of the experimental set‐up and key results, see Meir *et al*. (2015b) and Rowland *et al*. (2015c).

### Study specimens and drought sensitivity status

This study used six of the most common genera in the plots, which have been previously determined to be drought‐sensitive (*Manilkara*,* Eschweilera* and *Pouteria*) and drought‐resistant (*Protium*,* Swartzia* and *Licania*) through analysis of drought‐induced mortality rates (da Costa *et al*., 2010; Meir *et al*., 2015b; Rowland *et al*., 2015c). A genus was determined to be drought‐sensitive if it experienced 50% higher mortality and the death of at least two more individuals in the TFE than in the control plot (da Costa *et al*., 2010). This criteria were re‐applied by Rowland *et al*. (2015c) following 13 yr of experimental drought and the results were found to have remained consistent with the determination of da Costa *et al*. (2010). Henceforth, these genera are referred to simply as ‘sensitive’ or ‘resistant’ genera. Where possible, a single species was used to represent a genus (*Pouteria anomala* (Pires) T.D. Penn., *Manilkara bidentata* (A.DC.) A.Chev. and *Swartzia racemosa* (Benth.)), but more than one species was used where there were too few individuals in a species per plot: *Eschweilera* is represented by the species *Eschweilera coriacea* (DC.) S.A.Mori, *Eschweilera grandiflora* (Aubl.) Sandwith and *Eschweilera pedicellata* (Rich) S.A.Mori, *Licania* by *Licania membranacea* (Sagot ex Laness) and *Licania octandra* (Kuntze) and *Protium* by *Protium tenuifolium* Engl. and *Protium paniculatum* Engl. This approach was necessary to obtain sufficient numbers of trees within each genus and plot to enable a comparison, and has been adopted in other studies (Butt *et al*., 2008; van Mantgem *et al*., 2009). It is acknowledged that relevant interspecific differences do occur within a genus (Abrams, 1990), but in this study, variance among individuals within a genus was consistently less than variance among genera, as demonstrated by the difference between the percentages of variance explained by the random effects tree individual (ID) and genus (Gn) in Table 1.

**Table 1:** Proportions of variance of model components in percentage, total variance of transformed data and the conditional and marginal *r* ^2^

	$\Psi_{\pi }^{\mathrm{tlp}}$	$\Psi_{\pi }^{o}$	SWC	RWC^tlp^	ε	*C*
Variance (%)						
Fixed	30	32	4	13	13	10
Random						
ID	8	3	27	9	4	11
Gn	33	26	44	19	11	24
Residual	30	39	24	59	71	55
Total variance	0.1965	0.3060	0.0537	0.0090	0.3568	0.2809
*r* ^2^_conditional_	0.70	0.60	0.76	0.41	0.29	0.45
*r* ^2^_marginal_	0.30	0.32	0.04	0.13	0.13	0.10

The total variance used for calculating the percentages was determined using the product of the variance values derived from the models as per Nakagawa & Schielzeth (2013), and is, therefore, not identical to the ‘Total variance’ value listed in the table. Variables are turgor loss point ($\Psi_{\pi }^{\mathrm{tlp}}$_)_, osmotic potential at full turgor ($\Psi_{\pi }^{o}$_)_, saturated water content (SWC), relative water content at $\Psi_{\pi }^{\mathrm{tlp}}$ (RWC^tlp^), elastic modulus (ε) and capacitance (*C*), and the variance pertains to individuals (ID) from the six tropical rainforest genera (Gn) *Eschweilera*,* Licania*,* Swartzia*,* Manilkara*,* Pouteria* and *Protium*.

### Experimental protocol

#### Pressure–volume curves

To provide information on seasonal variability in PV parameters, measurements were carried out at the end of the dry season in November 2013 and the end of the wet season in May 2014, corresponding to periods of minimum and maximum soil water availability, respectively. The same sets of individuals were sampled in both periods, with the exception of the genus *Eschweilera* for which three additional individuals were measured on each plot in the dry season. Top‐canopy, fully sunlit branches were sampled, and after excision they were re‐cut under water and immediately transported back to the laboratory in water, where they were again re‐cut under water filtered to 0.2 μm, and then allowed to rehydrate overnight. Previous studies have demonstrated that rehydrating specimens before PV analysis can influence the results, particularly $\Psi_{\pi }^{o}$, which tends to increase (move closer to zero) as a result of very short‐term osmotic adjustment (Meinzer *et al*., 1986; Kubiske & Abrams, 1991; Yan *et al*., 2013; Meinzer *et al*., 2014). For two temperate zone species, Meinzer *et al*. (2014) showed that some PV parameters correlated strongly with the initial water potential (*r* ^2^ of 0.78 to 0.94 for the elastic modulus and turgor loss point (TLP), respectively) in the highly anisohydric species *Juniperus monosperma*, but this relationship was not found in the isohydric species *Pinus edulis*. However, because the purpose of this study was to compare changes in these and several other parameters (e.g. Rowland *et al*., 2015a,c) in response to long‐term drought and to seasonal differences in water availability, and not just initial water potential, full rehydration was employed to standardize starting conditions for all samples. Moreover, as there were 10 species in this study, presumably exhibiting different levels of isohydry, quantifying the degree of change with respect to initial water potential in each species would have been challenging given the field conditions. Leaves were selected that were fully expanded, mature and entirely unblemished, or had < 5% of their surface covered by epiphylls – lichens, fungi and mosses that colonize leaf surfaces. PV curves were obtained for a minimum of three leaves per genus per plot per season (one leaf per tree and nine leaves overall per sensitivity group) according to the ‘bench drying’ protocol described in Tyree & Hammel (1972). Briefly, as the leaf dried out over a period of 3–8 h, repeated measurements of leaf water potential (Ψ) and mass were taken using a Scholander pressure bomb (PMS Instruments Co., Corvallis, OR, USA) accurate to 0.05 MPa and mass balance accurate to 0.1 mg, respectively. After the final water potential measurement, the leaves were scanned to determine area using imagej software (Schneider *et al*., 2012) and then dried to constant mass in an oven at 70°C for > 48 h. The points were then plotted as 1/Ψ against leaf mass, enabling the calculation of the parameters osmotic potential at full turgor ($\Psi_{\pi }^{o}$; MPa), turgor loss point ($\Psi_{\pi }^{\mathrm{tlp}}$; MPa), saturated water content (SWC; the ratio of water mass to leaf dry mass in a fully saturated leaf; g g^−1^), relative water content at $\Psi_{\pi }^{\mathrm{tlp}}$ (RWC^tlp^; %), modulus of elasticity (ε; MPa) and hydraulic capacitance (*C*; mol MPa^−1^ m^−2^). Calculations of variables from PV curves were carried out according to Sack & Pasquet‐Kok (2011). We recognize that PV data analysis may contain a number of sources of error including the decision of which points to include to identify $\Psi_{\pi }^{\mathrm{tlp}}$. While it is very difficult to account for all possible error sources in a single analysis framework, we employed a maximum likelihood approach based on mixed effects modeling to avoid inflating degrees of freedom in nested samples and check normality assumptions (see ‘Statistical analysis of drought treatment effects on PV parameters’ for details of the statistical analysis).

#### Morphological traits

All samples for the tissue analysis were taken in the wet season. Small squares of leaf, *c*. 8 mm to a side, were taken from midway along the leaf between the midrib and the edge of the lamina and were sectioned using a hand‐held microtome (Euromex, Arnhem, Holland). Images of the sections were taken with a Moticam 2 digital camera on a Motic B3 microscope (Motic, Barcelona, Spain). A magnification of ×40 was used where the leaf was thin enough to view a whole section, from upper to lower cuticle, in one image. For thicker leaves it was sometimes necessary to use a magnification of ×10 to ensure that each tissue measurement was taken on a single ‘transect’, thus providing reliable proportional measurements. Where measuring all tissue layers on one image was not possible, multiple images were used per single leaf section – these values were only employed for absolute tissue measurements and were excluded from the analysis of proportional measurements. The values for each tissue thickness (abaxial epidermis (Ab), palisade (Pal), spongy mesophyll (SM) and adaxial epidermis (Ad)) for each tree are means taken from a single measurement from two leaves per tree.

The cavity volume of leaves (CV, otherwise referred to as leaf airspaces) was measured by subtracting the mass of fully hydrated leaves from the mass of the same leaves after perfusion with water. Branches were allowed to hydrate overnight and leaves were only used if adjacent leaves had a water potential higher than −0.2 MPa. The leaves were then weighed before being perfused with water at a pressure of 18 kPa for a minimum of 20 h and then reweighed. The risk of emboli forming in the petiole before perfusion was minimized by taking the initial weight with a small section of branch attached to the leaf. The petiole was then severed at its base with a razor blade under water filtered to 0.02 μm and attached to a silicon tube; the excised branch segment was then weighed and this was subtracted from the initial weight. Two leaves per individual were measured, all leaves being measured for area and dry mass. Cavity volume was expressed as volume per unit area (μm^3^ μm^−2^), which is equivalent to thickness per leaf section (μm) and so directly comparable to the other tissue thickness measurements.

The tissue measurements, cavity volumes and PV analysis were all carried out on different leaves to avoid the effects of one leaf manipulation influencing the others. Therefore, each set of measurements was averaged per individual tree to enable the correlation analysis to be performed. Both the tissue and the cavity volume measurements were only carried out in the wet season, but because genus was found to be the largest source of variance and because the seasonal effects were only found for SWC and RWC^tlp^, we pooled the results for the PV parameters across seasons to maintain the largest possible sample size.

### Statistical analysis of drought treatment effects on PV parameters

Results for the response of PV parameters to the drought treatment were analyzed using linear mixed effects models using the packages lme4 (Bates *et al*., 2015) and lmerTest in R (R Core Team, 2015). As the focus of this study was understanding sensitivity and resistance to drought and not the effect of taxon, genus and individual (tree) were included as nested random effects. Therefore, large differences between species within a genus would be represented by high variance in the random effect category ‘ID’ (tree individual), because the variance in the ID term groups the inter‐individual and inter‐species variance (Table 1). Models were initially constructed using all variables and interactions (e.g. treatment × season × sensitivity status), and were manually simplified by systematically removing nonsignificant variables and interactions. The best model (Table 2) was selected on the basis of the Akaike information criterion (AIC). The distribution of the data was assessed using the profile function as per Bates *et al*. (2015) and the data were transformed accordingly. The conditional and marginal *r* ^2^ values were calculated as per Nakagawa & Schielzeth (2013).

**Table 2:** Models used to describe variables measured in six tropical rainforest genera: *Eschweilera*,* Licania*,* Swartzia*,* Manilkara*,* Pouteria* and *Protium*

Response variable	Symbol	Units	Transformation	Model structure
Turgor loss point	Ψ_π_^tlp^	MPa	log(−1 × Y)	T × V × S
Osmotic potential at full turgor	Ψ_π_^0^	MPa	log(−1 × Y)	T × V × S
Saturated water content	SWC	g_water_ g^−1^_dry_mass_	log(Y)	T + S
Relative water content at TLP	RWC^tlp^	%	arcsin(Y/100)	S
Capacitance	*C*	mol Mpa^−1 ^m^−2^	log(Y)	T × V × S
Elastic modulus	ε	MPa	Y^0.34^	T × V × S

Model terms are as follows: T, treatment (through‐fall exclusion or control plot); V, drought vulnerability status (sensitive or resistant); S, season (dry or wet). In all models tree individual nested inside genus was a random effect used to adjust only the intercept.

### Regression analysis of leaf anatomy and PV parameters

The PV parameters were compared to the absolute and proportional thicknesses of leaf tissues using multiple linear regression in R (R Core Team, 2015). Of the PV parameters, $\Psi_{\pi }^{o}$ and $\Psi_{\pi }^{\mathrm{tlp}}$ represent conditions in the symplast, while the other parameters, SWC, RWC^tlp^, *C* and ε, represent the entire water‐occupied volume of the leaf. The elastic modulus, ε = d*P*/dRWC_leaf_, where *P* is the turgor pressure, can be calculated to represent only the symplast (and therefore the influence of turgor on cell wall expansion); however, bulk ε is used here to avoid errors arising from the extreme extrapolation of the PV curve to the *y* intercept needed to return ε for only the symplast (Andersen *et al*., 1991), and to minimize the impact of the necessary assumption that the apoplastic fraction remains constant throughout the PV analysis (Tyree & Richter, 1981). Therefore, the SWC, RWC^tlp^, *C* and ε all represent conditions of both the symplast and the cell walls, the ratio of which will vary between tissue layers depending on cell geometry and cell wall thickness. Absolute measurements of tissue thickness (Ab_abs_, Pal_abs_, SM_abs_ and Ad_abs_) represent volume per unit area (μm^3^ μm^−2^ = μm), and correlations of PV parameters with absolute measurements would indicate a functional link between the tissue type and the PV parameter. For example, a correlation between $\Psi_{\pi }^{o}$ and Pal_abs_, but not Pal_prop_ (palisade thickness as a proportion of leaf thickness), would indicate that a thicker palisade leads to or requires higher $\Psi_{\pi }^{o}$. However, correlations of PV parameters with proportional measurements of tissue thickness (Ab_prop_, Pal_prop_, SM_prop_ and Ad_prop_) would indicate which tissues are particularly influential in the overall leaf‐level value, and possibly are different from the leaf average. A significant relationship between a PV parameter and proportional tissue thickness might suggest that the properties of the tissue in question are important in determining the overall leaf‐level values.

Because $\Psi_{\pi }^{o}$ and $\Psi_{\pi }^{\mathrm{tlp}}$ are fundamental properties of the symplast which can become decoupled from cell volume through changes in cell size and cell wall thickness (Supporting Information Fig. S1), the same analysis was carried out by calculating the symplast volume of each tissue (Methods S1). The symplast volume was calculated using mean cell size and cell wall thickness, and by assuming that spongy mesophyll cells were spherical, palisade cells were cylindrical and epidermal cells were cuboids. It was not possible to measure cell wall thickness with sufficient accuracy because of the resolution of the images (S2), so mean cell wall thickness was derived from values presented by Buckley *et al*. (2015) for 14 species (*n *= 13 for spongy mesophyll). Given the additional error associated with the assumption of cell shape, the adoption of a mean cell wall thickness taken from other species, the reduction in the degrees of freedom because of the difficulty in measuring cell size accurately (mean df = 25 for tissue thickness and 17 for symplast volume) and the similarity in the results between the tissue thickness and the symplast analyses, the tissue thickness results are presented here while the alternative analysis is given in Table S1.

Tissue thicknesses, cavity volume and PV parameters were all averaged for each individual tree before performing the regression analyses. The cavity volume was subtracted from the total spongy mesophyll volume to give a value of water‐saturated spongy mesophyll volume, but the interaction between spongy mesophyll and cavity volume was analyzed for significance. This required the assumption that the cavity volume in the palisade layer was negligible compared with that in the spongy mesophyll, which was consistent with the images (Fig. S1). Absolute and proportional tissue thicknesses were modeled separately to highlight the different effects and to reduce correlation among independent predictors. Thus, the starting structure of the models was Y ~ Pal + Ad + Ab + SM × CV for both absolute and proportional measurements, where Y stands for the response variable and the sign ~ stands for ‘as a function of’. Models were simplified by sequentially removing the factors that did not contribute significantly to increase model log‐likelihood. At each simplification, successive models were compared using AIC values with a χ^2^ test. Variance inflation factors for all variables in the final models were found to be < 3, indicating very limited autocorrelation among independent variables.

## Results

### Hypothesis 1 (H_1_): Imposed drought vs seasonal effects

**Figure 1:**
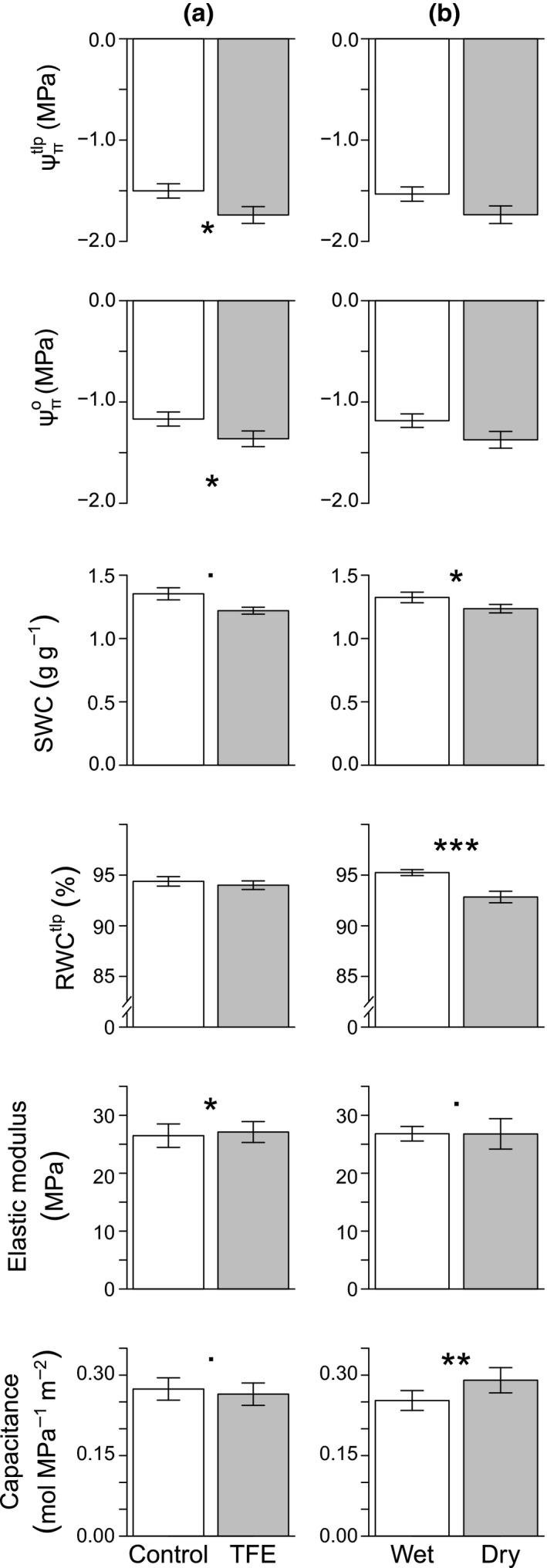
Comparison between seasonal and plot effects of pressure volume parameters in 44 tropical rainforest trees from six genera. (a) Comparison of plots. White bars, control plot; gray bars, through‐fall exclusion plot (TFE). (b) Comparison of seasons. White bars, wet season; gray bars, dry season. Bars display the mean ± 1 SE and significance is denoted by asterisks: *, < *P *=* *0.05; **, < *P *=* *0.01; ***, < *P *=* *0.001; *P *=* *0.05 < • < *P *=* *0.10. Annual rain in the drought plot is ≈90 mm per month, in the control plot is ≈180 mm per month, in the wet season (averaged between the TFE and control plot) is ≈210 mm per month and in the dry season is ≈60 mm per month. $\Psi_{\pi }^{\mathrm{tlp}}$_,_ turgor loss point; $\Psi_{\pi }^{o}$, osmotic potential at full turgor; SWC, saturated water content; RWC ^tlp^_is_, relative water content at $\Psi_{\pi }^{\mathrm{tlp}}$.

**Figure 2:**
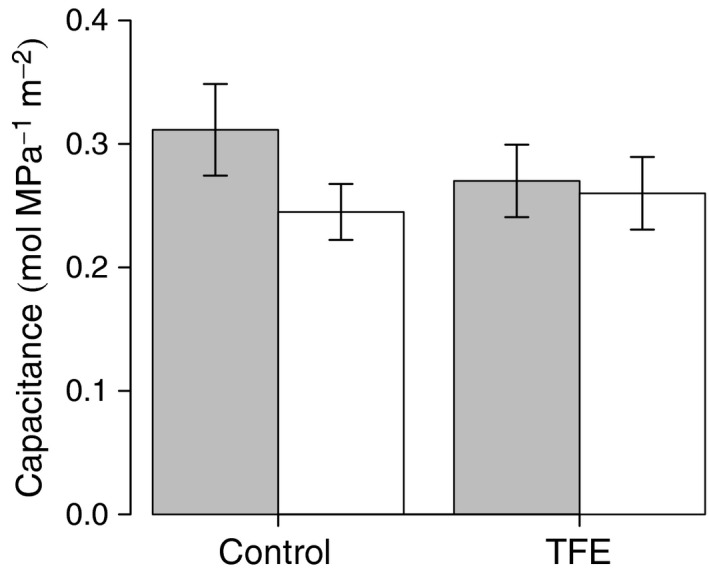
Plot (control and through‐fall exclusion (TFE)) and season (wet and dry) effects on hydraulic capacitance in 44 tropical rainforest trees from six genera. Both the seasonal effect (*P *=* *0.007) and the interaction between season and drought treatment (*P *=* *0.014) are significant. Gray bars, dry season; white bars, wet season. Bars display the mean ± 1 SE.

Water relation parameters varied greatly by season and treatment, with no common pattern existing across all parameters. Significant treatment effects were detected for $\Psi_{\pi }^{\mathrm{tlp}}$ (*P* = 0.041), $\Psi_{\pi }^{o}$ (*P* = 0.038) and ε (*P* = 0.030), while no significant effects were found for SWC, RWC^tlp^ and *C* (Table 3). Both $\Psi_{\pi }^{\mathrm{tlp}}$ and $\Psi_{\pi }^{o}$ (which were highly correlated; *r* ^2^ = 0.94), were lower (more negative) in the TFE compared to the control, while ε was larger in the TFE than in the control. By contrast, significant seasonal changes occurred for SWC, RWC^tlp^ and *C*, while no significant seasonal effects were detected in $\Psi_{\pi }^{\mathrm{tlp}}$, $\Psi_{\pi }^{o}$ and ε (Table 3; Fig. 1). Values of SWC and RWC^tlp^ were higher and those of *C* lower in the wet season. Thus, $\Psi_{\pi }^{o}$, $\Psi_{\pi }^{\mathrm{tlp}}$ and ε had stronger (long‐term drought) treatment than seasonal effects, consistent with H_1_. Trends in both *C* and ε were opposite to those expected from differences in water availability between season and treatment; *C* was highest in the dry season and in the control plot, with opposite trends for ε. Interactions between treatment and season were found only for capacitance, which showed no seasonal difference in the TFE, but an increase in the dry season in the control plot (Fig. 2).

**Table 3:** Probability values and coefficients for the fixed effects included in the mixed models listed in Table 2; factors with a dash were not included in the final model, and values where *P *<* *0.05 are in bold

Factor	$\Psi_{\pi }^{\mathrm{tlp}}$		$\Psi_{\pi }^{o}$		SWC		RWC^tlp^		ε		*C*	
	*P*	Coef.	*P*	Coef.	*P*	Coef.	*P*	Coef.	*P*	Coef.	*P*	Coef.
S	0.334	0.09	0.317	0.13	**0.015**	0.05	**< 0.001**	0.07	0.068	0.35	**0.007**	−0.42
T	**0.041**	0.25	**0.038**	0.33	0.068	−0.08	–	–	**0.03**	0.49	0.05	−0.38
V	0.599	−0.14	0.509	−0.20	–	–	–	–	0.876	−0.04	0.953	−0.02
S : V	**< 0.001**	−0.55	**< 0.001**	−0.71	–	–	–	–	0.156	−0.39	**0.044**	0.45
T : S	0.131	−0.21	0.089	−0.33	–	–	–	–	0.055	−0.53	**0.014**	0.55
T : V	0.084	−0.29	0.053	−0.41	–	–	–	–	**0.027**	−0.67	0.059	0.49
T : S : V	**0.007**	0.52	**0.004**	0.79	–	–	–	–	**0.044**	0.77	**0.018**	−0.74

Factors are season (S; dry or wet), treatment (T; through‐fall exclusion or control plot) and vulnerability status (V; drought‐sensitive or drought‐resistant). Variables are turgor loss point ($\Psi_{\pi }^{\mathrm{tlp}}$), osmotic potential at full turgor ($\Psi_{\pi }^{o}$_)_, saturated water content (SWC), relative water content at $\Psi_{\pi }^{\mathrm{tlp}}$ (RWC^tlp^), elastic modulus (ε) and capacitance (*C*).

### Hypothesis 2 (H_2_): Drought sensitivity status vs seasonal variation

**Figure 3:**
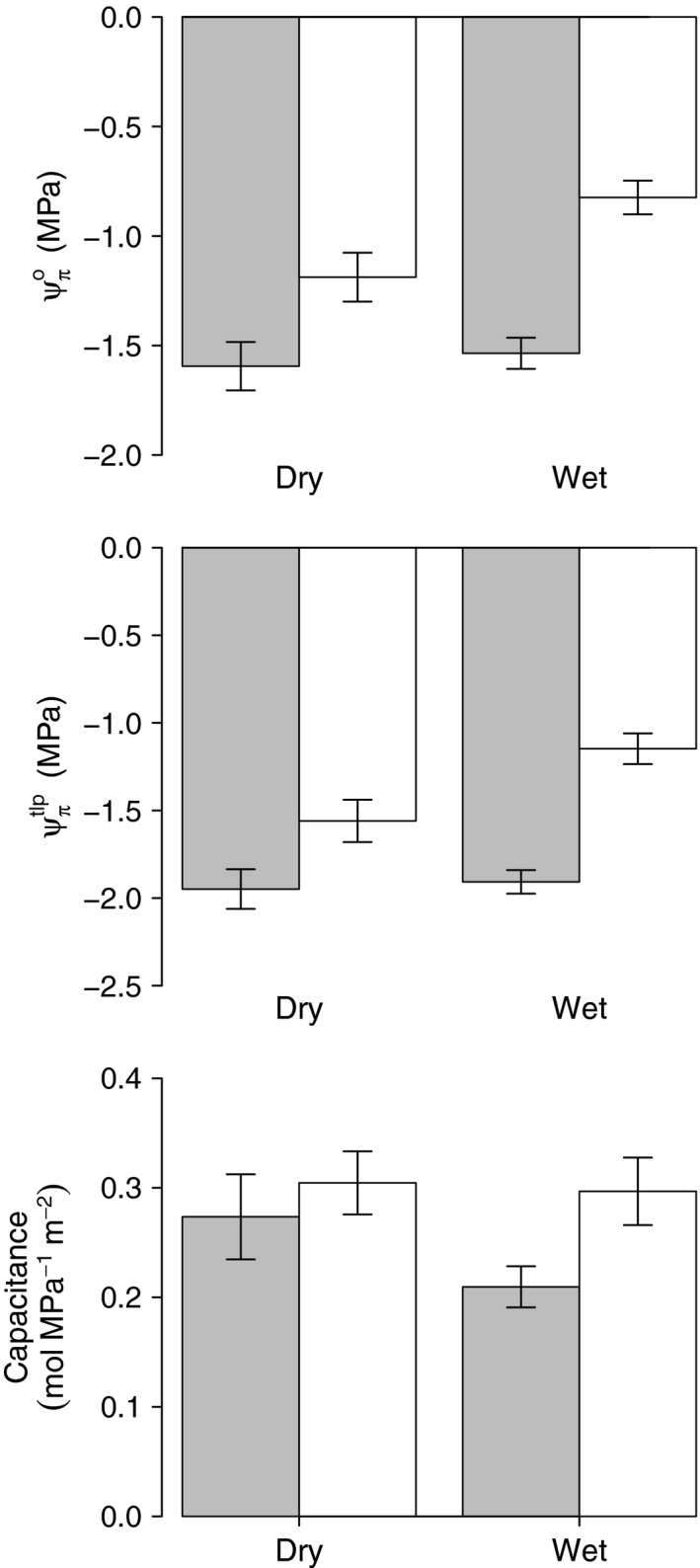
Season and drought sensitivity status effects for osmotic potential at full turgor ($\Psi_{\pi }^{o}$; *P *<* *0.001), osmotic potential at turgor loss point ($\Psi_{\pi }^{\mathrm{tlp}}$; *P *<* *0.001) and hydraulic capacitance (*P *=* *0.044) in 44 tropical rainforest trees from six genera. Gray bars, drought‐resistant species; white bars, drought‐sensitive species. Bars display the mean ± 1 SE.

Drought sensitivity status alone had no significant impact on any of the parameters, but there were significant interactions between sensitivity and season for $\Psi_{\pi }^{o}$ (*P* < 0.001), $\Psi_{\pi }^{\mathrm{tlp}}$ (*P *<* *0.001) and *C* (*P* = 0.044; Table 3; Fig. 3). In resistant species, $\Psi_{\pi }^{o}$ and $\Psi_{\pi }^{\mathrm{tlp}}$ showed little seasonal variation but in sensitive species both parameters were higher in the wet season. This is opposite to H_2_, that resistant genera would show greater seasonal variation. However, the reverse trend was evident for *C*, in which greater seasonal changes occurred in resistant species. Significant three‐way interactions occurred between treatment, season and sensitivity status for $\Psi_{\pi }^{\mathrm{tlp}}$ (*P* = 0.007), $\Psi_{\pi }^{o}$ (*P* = 0.004), ε (*P* = 0.044) and *C* (*P *=* *0.018), because of a large treatment effect among resistant species in the dry season, which was largely absent in the wet season and for the sensitive species.

### Variance in drought treatment analysis

The *r* ^2^_conditional_, showing the total amount of variance explained by the models, varied from 0.29 for ε to 0.76 for SWC (Table 1). The greatest proportion of explained variance in the mixed effects models was accounted for by the experimental (fixed) effects in $\Psi_{\pi }^{o}$ and ε but by random differences from genus to genus in the other variables. The variance attributed to individuals within a genus was typically a small proportion (3–11%) of total variance (with the exception of SWC: 27%), indicating that traits varied little among individuals within these genera. The modeled fixed effects accounted for between 4 and 32% of total variance, and were highest for $\Psi_{\pi }^{\mathrm{tlp}}$ and $\Psi_{\pi }^{o}$, at 30 and 32%, respectively.

### Hypothesis 3 (H_3_): PV traits and tissue correlations

**Figure 4:**
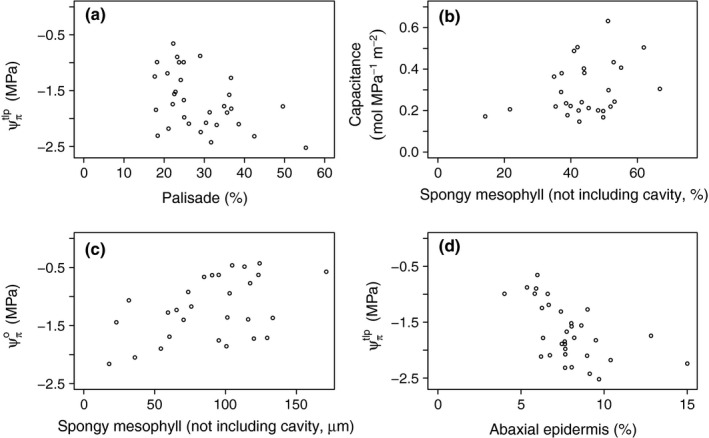
Relationships between pressure–volume parameters and tissue thickness in 28 tropical rainforest trees from six genera. The Pearson product–moment correlation coefficient for: (a) is *r *=* *−0.44, (b) is *r *=* *0.32, (c) is *r *=* *0.47, and (d) is *r *=* *−0.55. $\Psi_{\pi }^{\mathrm{tlp}}$ is turgor loss point and $\Psi_{\pi }^{o}$ is osmotic potential at full turgor.

Contrary to expectation, there was no correlation between $\Psi_{\pi }^{o}$ and either Pal_abs_ or Pal_prop_, but Pal_abs_ was significantly negatively correlated with $\Psi_{\pi }^{\mathrm{tlp}}$ and the relationship between Pal_prop_ and $\Psi_{\pi }^{\mathrm{tlp}}$ was marginally significant (Fig. 4a; Table 4). SM_prop_ correlated with *C* (Fig. 4b) and $\Psi_{\pi }^{o}$_,_ and, interestingly, SM_abs_ had highly significant positive correlations with $\Psi_{\pi }^{\mathrm{tlp}}$, $\Psi_{\pi }^{o}$ (Fig. 4c), SWC and RWC^tlp^. As SM_abs_ correlated strongly with leaf thickness (*R* ^2^ = 0.76; *P* << 0.001), they were employed in separate models to determine whether the correlations with SM_abs_ arose simply as a function of leaf thickness. Neither $\Psi_{\pi }^{\mathrm{tlp}}$ nor $\Psi_{\pi }^{o}$ correlated with leaf thickness, while both SWC and RWC^tlp^ did (*R *=* *0.48, *P *=* *0.003, and *R* ^2^ = 0.61, *P *<* *0.001, respectively) albeit less strongly than with SM_abs_. Ab_prop_ correlated with $\Psi_{\pi }^{\mathrm{tlp}}$ (Fig. 4d) and $\Psi_{\pi }^{o}$, but Ab_abs_ correlated only with $\Psi_{\pi }^{\mathrm{tlp}}$. However, Ad_abs_ correlated with $\Psi_{\pi }^{\mathrm{tlp}}$, $\Psi_{\pi }^{o}$, *C* and ε, but Ad_prop_ only correlated with RWC^tlp^. The absolute measurements of cavity volume did not correlate with any of the variables but significantly improved the strength of the models for $\Psi_{\pi }^{\mathrm{tlp}}$, SWC and *C*, while CV_prop_ only significantly improved the model for *C*. The models were initially performed with response variables transformed as in the mixed models; however, the transformation made little difference to the model results and so transformations were not used to facilitate interpretation of the model coefficients.

**Table 4:** Slope coefficients for linear regressions of pressure volume parameters in tropical rainforest trees against tissue thickness, expressed in either absolute (upper section) or proportional units (lower section)

	Tissue	Models					
		$\Psi_{\pi }^{\mathrm{tlp}}$ (MPa)	$\Psi_{\pi }^{o}$ (MPa)	SWC	RWC^tlp^ (%)	*C* (mol MPa^−1^ m^−2^)	ε (MPa)
Absolute tissue thickness (μm × 10^−3^)	SM_abs_	9.88***	9.31***	5.27***	46.90***	–	–
	Pal_abs_	−10.49*	−6.33	−5.74**	–	–	–
	Ab_abs_	−32.21*	−29.35 ·	–	–	–	433.90
	Ad_abs_	−30.83**	−27.03*	–	–	−7.07*	655.90**
	CV_abs_	92.01 ·	–	60.88 ·	–	30.88 ·	–
	***P*** **value**	**< 0.001**	**< 0.001**	**< 0.001**	**< 0.001**	0.056	**0.001**
	*R* ^2^_adjusted_	0.67	0.61	0.48	0.44	0.15	0.31
	df	21	22	24	26	24	31
Proportional tissue thickness	SM_prop_	1.62 **·**	2.33**	–	–	0.50 *	−34.07·
	Pal_prop_	−1.96 ·	–	−1.48**	–	–	–
	Ab_prop_	−8.69*	−8.10*	–	–	–	–
	Ad_prop_	–	–	–	−17.58**	–	–
	CV_prop_	–	–	–	–	0.60 ·	–
	***P*** **value**	**< 0.001**	**< 0.001**	**0.004**	**0.005**	0.059	**0.061**
	*R* ^2^_adjusted_	0.56	0.51	0.22	0.21	0.14	0.1
	df	21	22	30	30	25	26

Tissue parameters with a dash were not included in the final model. Significance is denoted by asterisks: *, < *P *=* *0.05; **, < *P *=* *0.01; ***, < *P *=* *0.001; *P *=* *0.05 < **· **< *P *=* *0.10, and significant values are in bold. The significance, *P*, proportion of explained variance, *R* ^2^, and the degrees of freedom, df, are given for each model. Variables are turgor loss point ($\Psi_{\pi }^{\mathrm{tlp}}$), osmotic potential at full turgor ($\Psi_{\pi }^{o}$), saturated water content (SWC), relative water content at $\Psi_{\pi }^{\mathrm{tlp}}$ (RWC^tlp^), elastic modulus (ε) and capacitance (*C*). Absolute measurements of tissue thickness are given in μm × 10^−3^, which gives units for the slope as e.g. ‘slope’ × 10^−3^ MPa μm^−1.^

Hypothesis 3, i.e. that Pal should correlate with $\Psi_{\pi }^{o}$, while SM should correlate with *C*, can be rejected in terms of there being no correlation between palisade thickness and $\Psi_{\pi }^{o}$, although the correlation between SM_prop_ and *C* may suggest that the spongy mesophyll plays a role in water storage. Interesting correlations that were not predicted include the negative correlation between the palisade thickness and $\Psi_{\pi }^{\mathrm{tlp}}$, the negative correlations between Ab_prop_ and both $\Psi_{\pi }^{\mathrm{tlp}}$ and $\Psi_{\pi }^{o}$ and the positive correlations between SM_abs_ and $\Psi_{\pi }^{\mathrm{tlp}}$, $\Psi_{\pi }^{o}$, SWC and RWC^tlp^.

## Discussion

This study reveals how leaf water relations in Amazonian rainforest trees respond to long‐term experimental drought and whether these responses are related to: (1) seasonal leaf water relations; (2) differential rates of drought‐induced mortality; (3) leaf tissue morphology. Overall, the studied trees, independent of drought‐sensitivity status, showed greater acclimation to the experimental soil moisture deficit than to seasonal variation in water availability, primarily via osmotic adjustments (H_1_). The designation of drought sensitivity of a species (based on mortality response; da Costa *et al*., 2010) was only important in these data with respect to differences in seasonal acclimation: drought‐sensitive species underwent greater levels of seasonal osmotic adjustment than resistant species (H_2_; Table 3), but a significant difference in the sensitivity status *per se* was not found. Lastly, palisade thickness did not correlate with osmotic potential at full turgor, but SM_prop_ did correlate with leaf hydraulic capacitance (H_3_; Table 4). Our data imply that caution is needed in ascribing acclimation capability to drought based on short‐term (seasonal) data: we demonstrate that tissue‐level water relation traits can acclimate to long‐term water stress, but that seasonal osmotic adjustment may not be an adaptive advantage in coping with extended drought stress.

### H_1_: Imposed drought vs seasonal response in $\Psi_{\pi }^{o}$, $\Psi_{\pi }^{\mathrm{tlp}}$ and ε

Consistent with H_1_, $\Psi_{\pi }^{o}$, $\Psi_{\pi }^{\mathrm{tlp}}$, and ε all showed a significant response to the drought treatment and no seasonal effect. Stable osmotic gradients, such as those between the symplast and apoplast, require energy to be created and maintained as they involve moving molecules up a gradient of osmotic potential (Nobel, 1999). Moreover, excessively high solute concentrations, as a result of dehydration, run the risk of causing membrane damage (Steponkus, 1984; Bryant *et al*., 2001). The cost and risk associated with increasing solute concentration are, therefore, likely to result in a physiological maximum solute concentration. The finding that $\Psi_{\pi }^{o}$ is significantly different between plots, but not seasons, indicates that the magnitude of seasonal osmotic adjustment does not represent a physiological limit for longer term water deficits and is therefore not a good indicator of a species’ capacity to cope with long‐term reduction in water availability. The higher ε in the TFE is consistent with the general negative correlation between ε and $\Psi_{\pi }^{o}$ (Niinemets, 2001; and Bartlett *et al*., 2012), and the combination of the changes in these two parameters contributes to drought resistance by creating a greater change in Ψ for a given amount of water loss, thus facilitating water uptake from drier soils without turgor loss (Bowman & Roberts, 1985). It is not known what determines the maximum capacity for adjustment in osmotic properties or the elastic modulus and, therefore, the adaptation of the trees in this study could not have been predicted without a long‐term experiment. The ability of trees to adapt to long‐term changes in water availability is fundamental to predicting how tropical forests are going to respond to climate change and, if overlooked, could lead to inaccurate projections of future vegetation–climate interactions.

### H_2_: Seasonal plasticity and drought sensitivity

Several studies have indicated that osmotic adjustment is linked to drought resistance (Kubiske & Abrams, 1991; Tschaplinski *et al*., 1998; Mitchell *et al*., 2008), suggesting that drought‐resistant species should show greater seasonal variation in osmotic traits (H_2_). In contrast to this expectation, it was the drought‐sensitive species that showed greater seasonal osmotic adjustment (Fig. 3), while the resistant species showed very little. The drought‐sensitive species had significantly higher (less negative) $\Psi_{\pi }^{o}$ and $\Psi_{\pi }^{\mathrm{tlp}}$ in the wet than dry season, which should, presumably, lead to lower maintenance costs than in the resistant species. On this basis, drought‐sensitive species might be expected to have lower respiration than the resistant species. However, there was no correlation between $\Psi_{\pi }^{o}$ and leaf dark respiration among these species (*P *=* *0.4; *R* ^2^ = 0.02; data not shown) and previous work has demonstrated that the leaves of the sensitive species in the drought plot had higher leaf dark respiration, especially in the dry season (Rowland *et al*., 2015c). Capacitance also showed an interaction between season and vulnerability status, but with a reverse trend to the osmotic parameters, in which the resistant genera showed seasonal variation while the sensitive genera showed little response (Fig. 3).The finding that most osmotic adjustment happened in the sensitive species may indicate that, rather than being an active strategy to reduce sensitivity to water stress, it may be an indirect result of another process. It is also worth stressing that no significant effect of the sensitivity status was found on leaf nonstructural carbohydrate concentrations and that this last parameter even increased slightly in the dry season in all species (Rowland *et al*., 2015c).

There was an apparent divide between the parameters in this study that responded significantly to the drought treatment, $\Psi_{\pi }^{o}$, $\Psi_{\pi }^{\mathrm{tlp}}$, and ε, and those that responded more to seasonality, SWC, RWC^tlp^ and *C* (Table 3). SWC, *C* and RWC^tlp^ have also been suggested to play a role in drought resistance (Kubiske & Abrams, 1991; Niinemets, 2001; Hao *et al*., 2008) but, in this case, their response to the experimental drought was not significant (*P *>* *0.05), despite their short‐term response to seasonal water availability. Given the mechanistic nature of the links between PV parameters, the disparity in responses between the two groups of traits may be seen as surprising. It is possible that the difference between the groups is caused by seasonal changes in cell wall properties; hence $\Psi_{\pi }^{o}$ and $\Psi_{\pi }^{\mathrm{tlp}}$ do not change seasonally, as they are properties of the symplast, while ε would be influenced only slightly by the changes in the water content of the cell walls. Another potential explanation is ontogenetic changes, whereby leaves of a similar age change systematically throughout the year. However, immature leaves were intentionally avoided and an analysis of variability in mean leaf area across seasons demonstrated that leaves were fully expanded (unpublished data). Therefore, it is concluded that, while seasonal differences alone were not significant in the osmotic parameters or ε, there were nonsignificant seasonal trends (Fig. 1) which led to significant variation in the other parameters.

### H_3_: Correlations between anatomical and water relation traits

It was hypothesized that the thickness of the palisade layer would correlate negatively with osmotic potential at full turgor (i.e. that leaves with thicker palisade would have more negative $\Psi_{\pi }^{o}$) and that the spongy mesophyll would correlate positively with capacitance. We found no evidence that the palisade thickness (calculated as either total or symplastic fraction) influenced leaf osmotic potential at full turgor and in this respect our data reject H_3_; however, the correlation of SM_prop_ with *C* suggests that the spongy mesophyll may affect leaf‐level capacitance (Table 4; Fig. 4b). The analysis of symplastic fractions (Table SI) yielded no correlations between capacitance and SM, perhaps arguing for a capacitive role of the apoplast of the SM. While neither of the palisade measurements correlated with $\Psi_{\pi }^{o}$, the correlation of Pal_abs_ and the weak correlation of Pal_prop_ with $\Psi_{\pi }^{\mathrm{tlp}}$ (*P* = 0.021 and *P* = 0.052, respectively; Table 4; Fig. 4a) could imply osmotic adjustment in the palisade layer in response to dehydration. Thus, it is unlikely that the osmotic potential of the palisade layer is significantly below that of the bulk leaf value when the leaf is hydrated (above $\Psi_{\pi }^{\mathrm{tlp}}$), but it is possible that solutes are generated in, or moved into, the palisade in response to leaf dehydration. These correlations disappeared when using the symplastic fractions of Pal and Pal_prop_, but the available degrees of freedom were drastically reduced for this analysis.

### Other correlations between anatomy and PV traits

The strong negative correlation between the proportional thickness of the abaxial epidermis and both $\Psi_{\pi }^{o}$ and $\Psi_{\pi }^{\mathrm{tlp}}$ (Fig. 4d; see also Table S1 for the symplastically adjusted values) implies that either leaves with low osmotic potentials benefit from having a thicker abaxial epidermis or that the abaxial epidermis has a lower $\Psi_{\pi }^{o}$ than the rest of the leaf (Mott, 2007). The latter hypothesis is in line with the findings of Buckley *et al*. (2015) that the upper and lower epidermal layers are hydraulically independent. Stomata close in response to a threshold leaf water potential (Brodribb *et al*., 2003), and thus by having an osmotic potential lower than the leaf average, turgor in the abaxial epidermis would be higher than the leaf average, enabling stomata to remain open when the epidermis is close to bulk leaf $\Psi_{\pi }^{\mathrm{tlp}}$. This strategy would be associated with anisohydric behaviors, which is consistent with recent findings from the same trees (unpublished data).

The absolute thickness of the spongy mesophyll appears to play an influential role in determining leaf PV values (Tables 4, S1). The strong correlation of SM_prop_ with $\Psi_{\pi }^{o}$ (Fig. 4c) could indicate that the SM has a higher osmotic potential (closer to 0) than the other tissues and/or that the structure of the SM compensates for the effects of low osmotic potential. The first possibility (higher osmotic potential, closer to 0) is consistent with the significant positive correlation between SM_prop_ and *C* (Table 4; Fig. 4b), although these results will have been influenced by two samples with particularly low SM_prop_, and there is also no correlation between the symplastic volume and *C* (Table S1). The second point, that the structure of the spongy mesophyll compensates for low (more negative) bulk leaf $\Psi_{\pi }$, supports the view that the spongy mesophyll offers a low resistance (high conductance) pathway for lateral hydraulic flow, in contrast to the palisade mesophyll (Wylie, 1946). Because water moves down a water potential gradient, flow can be increased by increasing either the gradient or the conductance according to the relation *F* = ∆Ψ × *K*, where *F* is flow rate and *K* is conductance. A thick spongy mesophyll, represented here without the cavity volume, can have large lateral connectivity (Fig. S2a; Wylie, 1946), potentially increasing hydraulic conductance within the leaf, and so reducing the need for low osmotic potential required for maintaining turgor with low water potentials.

### Variance accounted for by individual and genus

The percentage of variance accounted for by ID (individual tree within a genus; random effect) was low for most parameters, with the exception of SWC. By contrast, the variance accounted for by genus was relatively high (Table 1), indicating that the variation within a genus is lower than the variation among genera, and hence that there is some conservation of these parameters by taxonomic group. Bulk elastic modulus had the lowest variance among genera, suggesting convergence on a similar strategy regarding cell wall rigidity; conversely, SWC had high variance, suggesting divergence among genera in overall water content.

### Wider implications and summary

There is mounting evidence that hydraulic processes are fundamental to understanding drought‐induced tree mortality (Anderegg *et al*., 2012; Hartmann *et al*., 2015; Rowland *et al*., 2015a), and consequently there is increasing interest in how knowledge of hydraulic responses could inform ecosystem models. This study demonstrates that the six focal tropical tree genera can perform osmotic adjustment in response to long‐term (decadal‐scale) reductions in soil water availability over and above those associated with seasonal variation, and that seasonal osmotic adjustment does not act as an indicator of increased resilience to long‐term drought stress, and supports the hypothesis that different leaf tissues respond to hydraulic demands in different ways. While these findings only cover six genera, they suggest that, in contrast to those found in drier ecosystems (Kubiske & Abrams, 1991; Tschaplinski *et al*., 1998; Mitchell *et al*., 2008), maintaining osmotic homeostasis may be a more successful drought resistance strategy than relying on osmotic adjustment in tropical rainforest communities.

Results such as these are vital for understanding how we can predict plant responses under future water stress in tropical forests, for which further empirical understanding of both long‐ and short‐term responses to drought conditions is urgently needed.

## Author contributions

O.B. and M.M. led, and O.B. implemented the study. O.B., M.M. and P.M. designed the research. P.M. and A.C.L.C. conceived and implemented the experiment, with L.F. enabling data collection. O.B., L.R., A.A.R.O. and B.C. collected the data, and S.S.V. provided equipment. O.B. analyzed the data with M.M. O.B. wrote the paper with L.R., M.M., P.M. and A.N.

## Supporting information

Please note: Wiley Blackwell are not responsible for the content or functionality of any supporting information supplied by the authors. Any queries (other than missing material) should be directed to the *New Phytologist* Central Office.

**Fig. S1** Relationships between the symplastic fraction of the spongy mesophyll, and the spongy mesophyll symplast volume per unit area, and spongy mesophyll thickness.
**Fig. S2** Transmission light microscope images of leaf sections of *Pouteria anomala*,* Eschweilera coriacea* and *Swartzia racemosa*.
**Table S1** Slope coefficients for correlations of PV parameters against the symplastic fraction of tissue thickness
**Methods S1** Regression analysis of symplastic tissue volume vs leaf water relations.
