# Supplementary material for: Plasticity in leaf‐level water relations of tropical rainforest trees in response to experimental drought
Source: New Phytol. 2016 Mar 22;211(2):477–88. doi: 10.1111/nph.13927 (PMC5071722; doi:10.1111/nph.13927)
Supplement: Supplementary file 1 — Fig. S1 Relationships between the symplastic fraction of the spongy mesophyll, and the spongy mesophyll symplast volume per unit area, and spongy mesophyll thickness. Fig. S2 Transmission light microscope images of leaf sections of Pouteria anomala, Eschweilera coriacea and Swartzia racemosa. Table S1 Slope coefficients for correlations of PV parameters against the symplastic fraction of tissue thickness Methods S1 Regression analysis of symplastic tissue volume vs leaf water relations. [file NPH-211-477-s001.pdf]

New Phytologist

***New Phytologist* Supporting Information**

Article title: **Plasticity in leaf-level water relations of tropical rainforest trees in response to experimental drought**

Authors: Oliver Binks, Patrick Meir, Lucy Rowland, Antonio Carlos Lola da Costa, Steel Silva Vasconcelos, Alex Antonio Ribeiro de Oliveira, Leandro Ferreira, Bradley Christoffersen, Andrea Nardini and Maurizio Mencuccini

Article acceptance date: 07 February 2016

The following Supporting Information is available for this article:

Fig. S1 Relationships between (a) the symplastic fraction of the spongy mesophyll, and (b) the spongy mesophyll symplast volume per area with spongy mesophyll thickness.

Fig. S2 Transmission light microscope images of leaf sections of (a) *Pouteria anomala*, (b) *Eschweilera coriacea* and (c) *Swartzia racemosa*.

Table S1 Slope coefficients for correlations of PV parameters against the symplastic fraction of tissue thickness.

Methods S1 Regression analysis of symplastic tissue volume vs leaf water relations.

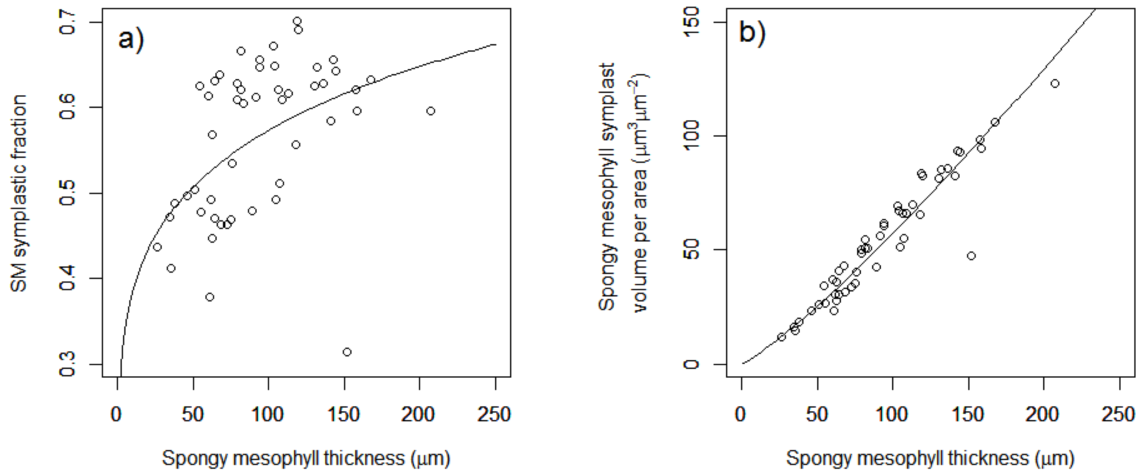

Fig. S1 Relationships between (a) the symplastic fraction of the spongy mesophyll, and (b) the spongy mesophyll symplast volume per area with spongy mesophyll thickness. (a) $y = 0.250966 \times x^{0.1767}$, $R^2 = 0.19$ and $P = <0.001$. (b) $y = 0.250966 \times x^{1.1767}$, $R^2 = 0.92$ and $P << 0.001$.

Table S1 Slope coefficients for regression of pressure volume parameters against the symplastic fraction of tissue thickness, expressed in either absolute (upper section) or proportional units (lower section).

		Models					
	**Tissue**	Ψ_{π}^{tlp} (MPa)	Ψ_{π}^{o} (MPa)	SWC	RWC (%)	C (mol MPa^{-1} m^{-2})	ε (MPa)
**Adjusted tissue thickness (μm × 10^{-3})**	SM_{symp}	**19.90****	**17.39****	**7.58****	**78.60*****	2.76 ·	-
	Pal_{symp}	-	-	-	-	-	-
	Ab_{symp}	**110.26****	**-102.90****	-	-	-9.41	**1150.70***
	Ad_{symp}	-	-	-	-	-	**821.70***
	***P* Value**	**0.002**	**0.002**	**0.002**	**<<0.001**	0.181	**0.001**
	$R^2_{adjusted}$	0.56	0.56	0.32	0.51	0.11	0.47
	df	13	13	22	22	13	18
**Symplast thickness as a proportion of total leaf thickness**	SM_{symp}/T_{leaf}	**2.80***	**2.88***	**1.48***	**20.26****	0.35	-21.36
	Pal_{symp}/T_{leaf}	-2.08	-	-1.86 ·	-	-	-
	Ab_{symp}/T_{leaf}	**-19.97***	**-19.03***	-	-	-	**389.34***
	Ad_{symp}/T_{leaf}	-	-	-	-	-	-
	***P* Value**	**0.005**	**0.003**	**0.010**	**0.001**	0.267	**0.025**
	$R^2_{adjusted}$	0.54	0.53	0.29	0.37	0.01	0.43
	df	12	13	21	22	22	13

Tissue parameters with a dash were not included in the final model. Significance is denoted by asterisks: *, < P=0.05; **, < P=0.01; ***, <P=0.001; P=0.05 < · < P=0.10, and significant values are in bold. The significance, P, proportion of explained variance, R^2, and the degrees of freedom, df, are given for each model. Variables are turgor loss point (Ψ_{π}^{tlp}), osmotic potential at full turgor (Ψ_{π}^{o}), saturated water content (SWC), relative water content at Ψ_{π}^{tlp} (RWC^{tlp}), elastic modulus (ε) and capacitance (C). Absolute measurements of tissue thickness are given in μm × 10^{-3}, which gives units for the slope as e.g. 'slope' × 10^{-3} MPa μm^{-1}.

Methods S1 Correlation analysis of symplastic tissue volume vs leaf water relations. Osmotic potential at full turgor and turgor loss point pertain only to the symplast volume of cells. If differences exist in these parameters between tissue types within the leaf one might expect a correlation to exist between the thickness of the particular tissue with the osmotic parameter. However, such relationship, if one exists, could become decoupled from tissue thickness due to changes in cell size and apoplastic fraction. Therefore, a separate analysis was performed to examine the correlations without the volumetric apoplastic fraction.

Cell sizes were measured by analysing sections of leaf, *c*. 10 μm thick, on transmission light microscope images using ImageJ software. Spongy mesophyll cells were assumed to be spherical and represented by a single measurement of diameter, palisade mesophyll cells were assumed to be cylindrical and were represented by measurements of length and diameter, and epidermal cells were assumed to be cuboids, square in paradermal section, represented by measurements of length and thickness. Five measurements of each were made per leaf section, and each tree was represented by two leaves, one section from each. All measurements were averaged per tree for the correlation analysis with the PV parameters.

For the correlation analysis (see 'Regression analysis of leaf anatomy and PV parameters' in the Materials and Methods section for further details) cell volumes were calculated based on the measurements described above. The apoplastic volume of each cell was calculated using average cell wall thicknesses taken from Buckley *et al*. (2015). The values were means taken from 14 species (13 for spongy mesophyll) and are as follows in μm ± 1 SE: $Ad_{cell\_wall} = 1.87 \pm 0.16$, $Pal_{cell\_wall} = 1.15 \pm 0.09$, $SM_{cell\_wall} = 1.31 \pm 0.14$, $Ab_{cell\_wall} = 1.71 \pm 0.15$. Thus, the symplastic fraction was determined by subtracting the apoplastic volume from the total cell volume and dividing it by total cell volume. The spongy mesophyll, for example, would be:

V_{sm_T} = Volume of spongy mesophyll cell (μm^3)
V_{sm_s} = symplast volume of SM cell (μm^3)
F_{sm_s} = symplastic fraction of SM cell

r_{sm} = radius of SM cell (μm)

T_{sm_cw} = Thickness of the cell wall (μm)

$V_{sm\_T} = 4/3\pi r_{sm}^3$

$V_{sm\_s} = 4/3\pi(r_{sm} - T_{sm\_cw})^3$

$F_{sm\_s} = V_{sm\_s} / V_{sm\_T}$

As all of the tissue volumes in this paper are normalised by leaf area ($\mu m^3\ \mu m^{-2} = \mu m$), multiplying the thickness of tissue by the symplastic fraction gives the 'thickness' of symplast i.e. the volume of symplast per area:

$SM_{abs} \times F_{sm\_s} = SM_{symp}$ (μm)

Because this was just an analysis of symplast volume, the cavity volume was not analysed in these models. Otherwise, the analysis was conducted in exactly the same way as the analysis of the tissue thickness. Therefore, the starting structure of the models, using SM as an example, was:

$Y \sim Ad_{symp} + Pal_{symp} + SM_{symp} + Ab_{symp}$

To make this analysis correspond to the analysis of tissue thickness, the symplastic thickness of each tissue was also found as a proportion of total leaf thickness. Proportional measurements were not found by summing the fractional symplastic contribution because this resulted in a high degree of interdependence between values. Thus, $SM_{prop\_symp} = SM_{symp}$ / leaf thickness, not $SM_{symp} / (Ad_{symp}+Pal_{symp}+SM_{symp}+Ad_{symp})$.

Results and Discussion

Mean cell volume of all tissue layers increased significantly with tissue thickness. This was expected in the case of the epidermal layers which are one cell thick, and for the palisade which was often one cell thick (Fig. S2), but not expected for the SM ($R^2 = 0.16$, $P = 0.002$). Because SM cells were assumed to be spherical, the surface area to volume ratio was expected to decrease

non-linearly with volume and, therefore, the relationship between symplastic fraction and SM thickness was also predicted to be non-linear (Fig. S1).

The analysis of symplastic volumes with PV parameters provided results similar to those of the tissue thickness (compare Table S1 with Table 4). The absolute values of spongy mesophyll correlate with Ψ_{π}^{tlp}, Ψ_{π}^{o}, SWC and RWC^{tlp} in both analyses, although in the symplastic analysis the proportional measurements also correlate with Ψ_{π}^{tlp}, SWC and RWC^{tlp}. Perhaps the biggest difference between analyses is that neither the absolute or proportional measurements of the palisade correlate with anything. As the analysis of tissue thickness combines the apoplast and symplast, it is possible that disparity between the two analyses (symplast vs thickness) indicates a functional role for the apoplast. However, the results of the symplast analysis must be interpreted with caution due to the assumptions (listed below) made to derive symplast volume and to the reduced degrees of freedom of the analysis.

Assumptions required to determine symplast volume:

- Cells accurately represented by designated shape e.g. SM cell is spherical, palisade is cylindrical, epidermal cells are cuboid.
- Cell walls are a comparable thickness to the 14 species measured by Buckley (2015)
- Cuticle accounts for negligible proportion of leaf thickness.

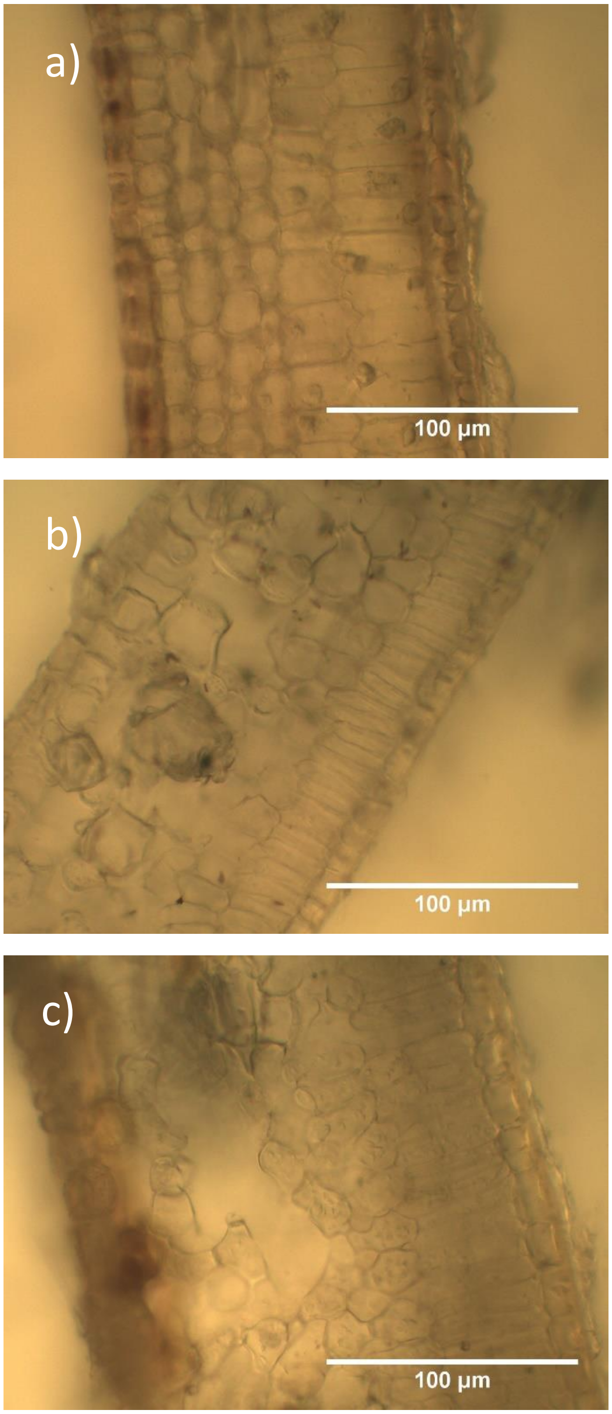

Fig. S2 Transmission light microscope images of leaf sections of (a) *Pouteria anomala*, (b) *Eschweilera coriacea* and (c) *Swartzia racemosa*. The pictures show densely packed palisade mesophyll layers with very little air space, and varying amounts of air space in the spongy mesophyll layer.

References

Buckley TN. 2015. The contributions of apoplastic, symplastic and gas phase pathways for water transport outside the bundle sheath in leaves. *Plant, Cell & Environment* **38**: 7–22.

Buckley TN, John GP, Scoffoni C, Sack L. 2015. How does leaf anatomy influence water transport outside the xylem? *Plant Physiology* **168**: 1616–1635.
